# The Association between Parental Involvement Behavior and Self-Esteem among Adolescents Living in Poverty: Results from the K-CHILD Study

**DOI:** 10.3390/ijerph17176277

**Published:** 2020-08-28

**Authors:** Satomi Doi, Aya Isumi, Takeo Fujiwara

**Affiliations:** 1 Department of Global Health Promotion, Tokyo Medical and Dental University (TMDU), Tokyo 113-8510, Japan; isumi.hlth@tmd.ac.jp (A.I.); fujiwara.hlth@tmd.ac.jp (T.F.); 2 Japan Society for the Promotion of Science, Tokyo 102-0083, Japan

**Keywords:** child poverty, self-esteem, parental involvement, parental interactions with child, parental care for child’s physical health, Japan

## Abstract

It is not yet known why some adolescents living in poverty show high self-esteem, while others do not. Parental involvement may be an important determinant to promote self-esteem among adolescents living in poverty. The aim of this study is to explore better parenting involvement behavior to promote self-esteem among adolescents living in poverty. Participants included fifth-, eighth-, and 11th-grade students living in Koichi prefecture, Japan. The participants were part of the Kochi Child Health Impact of Living Difficulty (K-CHILD) study, in 2016 (*n* = 10,784). Participants completed a questionnaire with questions about socioeconomic status and 14 parental involvement behaviors, including 9 specific kinds of parental interactions with their child (e.g., talking about school life), and 5 elements related to parental care for their child’s physical health (e.g., access to health care). The numbers of parental involvement behaviors, parental interactions with their child, and parental care for their child’s physical health were treated as continuous and quartile, to see the association. Overall, the study showed that the larger the number of parental involvement behaviors, the higher the self-esteem score of their off-spring (*p* < 0.01) among both adolescents living in poverty and not living in poverty, in which interaction between poverty and parental involvement behaviors was not significant. Both parental interaction with their child and parental care for their child’s physical health were associated with higher self-esteem, in which parental interaction with their child had a larger effect than parental care for their child’s physical health. To empower adolescents in poverty, caregivers need to provide both parental interaction with the child and parental care for the child’s physical health.

## 1. Introduction

The Organization for Economic Co-operation and Development (OECD) reported that the worldwide child poverty rate in 2015 was 13.4% [1]. The adverse impacts of child poverty have been well-documented: eczema [2], wheezing [3], a decline in pulmonary function [4], dental caries [5,6,7,8], suspected autism spectrum disorders [9], and low uptake of vaccinations [10,11,12,13]. Furthermore, it has been found that there are long-term impacts of child poverty, such as higher functional disability [14], depression [15], and dementia [16,17] among older adults who spent their childhood living in poverty.

Self-esteem among adolescents in poverty is a key element in breaking the cycle of poverty [18,19]. Self-esteem is referred to as “an individual’s subjective evaluation of her or his worth as a person” [20]. That is, children living in poverty may consider themselves to be worthless, and may be less likely to have self-care ability [21,22,23], which induces poor academic performance [24,25,26], poor health, such as mental health problems [27,28,29], behavioral problems [30,31], and physical health problems [31]. Finally, poor children are more likely to remain poor throughout adulthood, which is known as “the cycle of poverty” [32,33].

To break this poverty cycle, the factors that promote self-esteem in poor children must be elucidated. According to previous studies, parenting is a critical factor to promote self-esteem in children living in poverty. Previous studies, using a sample of families living in deprived areas, showed that the association between poverty and a child’s low self-esteem was mediated by poor quality of parenting [34,35,36]. For example, Conger et al. [34] which was a cross-sectional study of 205 families in the rural Midwest, indicated that the inverse association between poverty and self-confidence among boys was mediated by parenting style (i.e., involved, warm, low hostility, and discipline). Another longitudinal study of 674 families originally from Mexico also found that economic hardship had an impact on low self-esteem in children via parental warmth and monitoring [35]. Yoder and Hoyt [36] which was a cross-sectional study of 501 families in the United State showed that poverty was associated with parental depressive symptoms that increased hostile behavior and physical abuse, which had relationship with lower self-esteem in children. 

There are programs for caregivers living under the poverty line to improve parenting practices [37,38,39]. However, these programs have problems with rates of non-completion, approximately 50–60% of participants drop out [40]. Furthermore, these programs cannot be undertaken by all caregivers living in poverty due to budget constraints, and provider and caregiver motivation. Japan, in particular, lacks sufficient resources to improve the parenting practices of caregivers living in poverty. Therefore, a public health approach may be helpful to improve parental practices in as many caregivers as possible. 

Although the previous studies have shown strong evidence of the association between the quality of parenting practices (i.e., parenting style) and child’s self-esteem, the impact of quantity of parental involvement behaviors is also critical in a public health approach. Countable parental involvement behaviors such as parental interaction with a child (e.g., talking about school life) or parental care for a child’s physical health (e.g., preparing breakfast) may be helpful to provide key messages to caregivers and to assess parenting practices in a public health approach. Especially, focusing on positive parental involvement behavior makes caregivers change their behavior rather than trying to decrease negative parental involvement behavior. To date, it is unclear whether a larger number of parental involvement behaviors are associated with a higher level of self-esteem. We hypothesize that large numbers of parental involvement behaviors would promote self-esteem among children living in poverty.

Parental involvement behaviors can be divided into parental interaction with the child, and parental care for the child’s physical health. Previous studies indicate that parent-child direct interaction (i.e., parental interaction with child) helps reduce behavioral problems in children [41] and improve the efficacy of behavioral treatments such as parent-child interaction therapy in child mental health outcomes [42,43]. Previous studies also show the association between preparing a meal for a child [44,45], which indicates parental care for the child’s physical health, and the child’s mental health. However, it is unknown which aspects of parental involvement behaviors show a stronger association with self-esteem among children living in poverty.

The current study used population-based data from the Kochi Child Health of Living Difficulty (K-CHILD) study which examined the health and living environment of children in all public elementary schools (first and fifth grades), junior high schools (eighth grade), and high schools (11th grade), in Kochi prefecture, Japan. It was feasible to examine the association between child poverty and self-esteem in adolescents (i.e., fifth-, eighth-, 11th-grade students) because Kochi prefecture is a socioeconomically disadvantaged area (e.g., in 2015, the total rate of children coming from households receiving public assistance, child institutions, or single-parent homes was 12.4%, which was higher than Japan’s average of 8.0% in the same year [46]). 

The aim of this study is to explore better parental involvement behavior to promote self-esteem among adolescents living in poverty. In this study, we examine the association between the number of parental involvement behaviors and self-esteem among adolescents living in poverty and those not living in poverty. Moreover, we compare the association of two aspects of parental involvement behaviors (i.e., parental interactions with the child and parental care for a child’s physical health) with the amount of self-esteem among adolescents living in poverty and not living in poverty. We focus on positive and wide range of parental involvement behaviors, which might be helpful provide key messages to caregivers in a public health approach. 

## 2. Materials and Methods 

### 2.1. Participants

We used data from a population-based study of children, known as the Kochi Child Health Impact of Living Difficulty (K-CHILD) study (*n* = 23,750) in 2016, which examined the living environment and health of first-graders and fifth graders in all elementary schools, second graders in all junior high schools (i.e., eighth grade), and second graders in all high schools (i.e., 11th grade) in Kochi prefecture, Japan. Kochi prefecture is located on Shikoku Island, in southwest Japan. It has a population of 704,546 people and is known for fishing, agriculture, and forestry. (For details of the K-CHILD profiles see [47,48].

**Figure 1:**
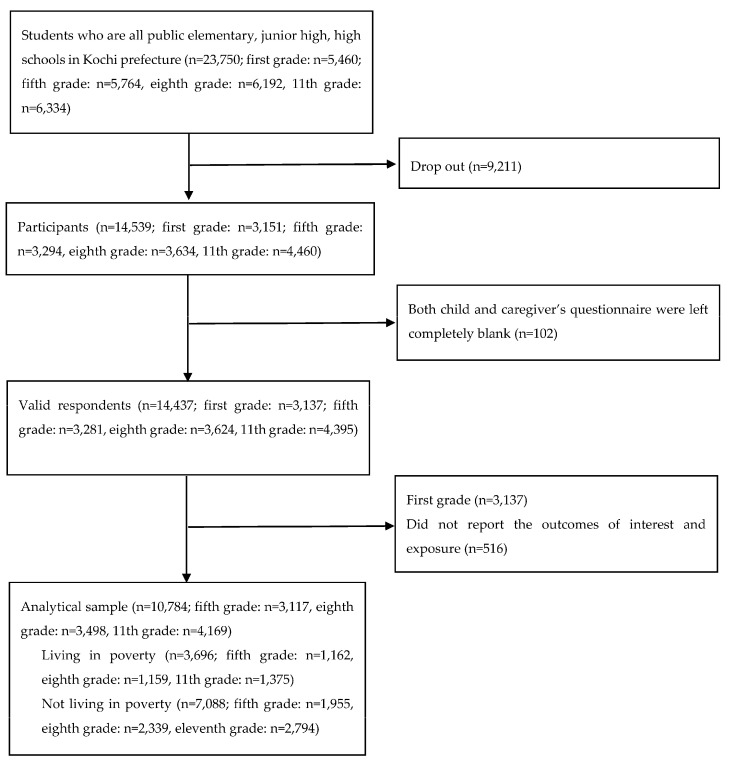
Requirement flow chart.

In this analysis, we used data only from adolescents (*n* = 10,784), excluding respondents who did not report the outcomes of interest (i.e., self-esteem) (*n* = 516) (Figure 1).

### 2.2. Measurements

#### 2.2.1. Child Poverty

In the current study, child poverty is defined as an adolescent who falls into any one of the following categories: (1) annual household income less than 3,000,000 yen; (2) household has material deprivation; (3) family lacks the capacity to pay for the cost of one or more types of essential utilities. We used this definition based on previous studies [19,49,50,51].

#### 2.2.2. Parental Involvement Behaviors

There are 14 “parental involvement behaviors” which are broken down into 9 elements related to “parental interaction with a child”, and 5 elements related to “parental care for child’s physical health.” Caregivers were asked to rate the frequency in which they interacted with their children while doing the following activities: (1) helping child study, (2) talking about school life, (3) talking about news, (4) playing with a child (physical activity), (5) talking about TV shows with child, (6) cooking with child, and (7) going out with a child (with response items of 1 (almost every day), 2 (3–4 times a week), 3 (1–2 times a week), 4 (1–2 times a month), and 5 (rarely)). The caregivers also rated the following 2 behaviors, (8) the frequency of talking about child’s future (with response items of 1 (frequently), 2 (sometimes), 3 (rarely), and 4 (never)), (9) the experience of hosting events for a child (i.e., giving a birthday party, taking a yearly family trip, and giving a present for Christmas or money at New Year’s) (with response items of yes or no). 

In terms of parental care for a child’s physical health, access to health care was assessed by the following 2 questions: (1) experience of not bringing the child to a doctor or hospital when they were sick (with response items of yes or no), and (2) history of routine vaccinations (with response items of yes, no, or unknown). Passive smoking was assessed as (3) maternal smoking in front of a child and (4) paternal smoking in front of a child (with response items of—always, sometimes, or never). Caregivers also rated cooking behaviors: (5) the frequency of cooking for a child (with response items of 1 (almost every day), 2 (4–5 days a week), 3 (2–3 days a week), 4 (a few days a month), and 5 (rarely)).

For aggregated measurements of parental involvement, we counted the number of the above behaviors in which the parents were involved. The factor analysis showed one factor (Eigenvalue was 1.75). The score of the parental involvement behaviors, which ranges from 0 to 14, was calculated by adding the number of parental involvement behaviors, as follows: helping child study (0 = less than once a week, 1 = once or more a week), talking about school life (0 = less than five times a week, 1 = almost every day), talking about news (0 = less than three times a week, 1 = three or more times a week), playing with child (0 = rarely, 1 = once or more a month), talking about TV shows with child (0 = less than five times a week, 1 = almost every day), cooking with child (0 = twice or less a month, 1 = once or more a week), going out with child (0 = twice or less a month, 1 = once or more a week), talking about child’s future (0 = sometimes or less, 1 = frequently), hosting events for child (0 = no, 1 = yes), experience of not bringing the child to a doctor or hospital when they were sick (0 = yes, 1 = no), history of routine vaccinations (0 = no/unknown, 1 = yes), maternal smoking in front of child (0 = smoking in front of child, 1 = never smoking/smoking, but not in front of child), paternal smoking in front of child (0 = smoking in front of child, 1 = never smoking/smoking, but not in front of child), and cooking for child (0 = five days or less a week, 1 = almost every day). The Cronbach’s alpha for the measurement of parental involvement behaviors was 0.62. 

#### 2.2.3. Self-Esteem

Adolescents assessed their self-esteem using one of the subscales from the Japanese version of Children’s Perceived Competence Scale [52], which was developed based on the Perceived Competence Scale for Children [53]. In this study, we used 9 items related to self-esteem (e.g., “Are you satisfied with the way you are now?” and “Do you think you have few good points?”) rated on a scale of 1 (*no*) to 4 (*yes*). One question, “Are you always worried about whether or not you will fail?” was removed from the survey as a high total score denoted a high level of self-esteem. The Cronbach’s alpha for the scale was 0.88 in this study.

#### 2.2.4. Covariates

Caregivers were asked about their relationship with their children (mother, father, grandparent, or others), child’s sex (male or female), maternal age, marital status (married, unmarried, divorced, or widowed), and whether the child participant had an older sibling (yes or no) or younger sibling (yes or no) as basic demographics. The caregiver’s childhood socioeconomic status was assessed using an indicator for economic difficulties in childhood (yes or no), along with a question about maternal education level (junior high school, high school dropout, high school, technical college, junior college, college dropout, college, graduate college, other, or unknown). Caregivers also rated their psychological distress using the Japanese version of the Kessler 6, K6; [54] (The Cronbach’s alpha was 0.90 in this study) on a scale of 1 (*all of the time*) to 5 (*none of the time*), which was categorized into three groups (0–<5, 5–<13, and 13+) and rated their relationship with their neighborhood on a scale of 1 (very close), 2 (close), 3 (have relationship, but not close), or 4 (no relationship), which was categorized into two groups (high (very close/close) and low (have a relationship, but not close/no relationship).

### 2.3. Ethics

This study was approved by the Ethics Committee of the Tokyo Medical and Dental University (M2017-243).

### 2.4. Statistical Analysis

First, we compared the differences in basic demographics, caregiver’s childhood socioeconomic status, and caregiver’s other possible confounders between the participants not living in poverty and living in poverty using a *t*-test and a chi-squared test. 

Second, we also compared the differences in parental involvement behaviors between the participants not living in poverty and living in poverty using a chi-squared test.

Third, a multivariate linear regression analysis was conducted to examine the association between the total number of parental involvement behaviors and self-esteem of adolescents living in poverty and those not living in poverty. After estimating a crude model, basic demographics (i.e., child sex, grade, respondent, maternal age (<40 years, 40 years–<50 years, and 50 years+), marital status (married, and unmarried/divorced/widowed), having an older sibling, and having a younger sibling), and maternal childhood socioeconomic status (i.e., economic difficulties in childhood, maternal economic difficulties in childhood, maternal education (high school or less, some college, college or more, and other/unknown)), and all 34 municipalities in Kochi prefecture were adjusted for potential confounders (Model 1). In Model 2, the caregiver’s psychological distress (K6) and relationship with their neighborhood were added. Additionally, we created categorical variable scores of parental involvement behaviors. We defined the four quantile groups and performed a multivariate linear regression analysis.

Fourth, in addition to parental involvement behaviors, we performed multivariate linear regression analyses to examine the associations of the total number of “parental interaction with their child” and “parental care for child’s physical care” with self-esteem of adolescents living in poverty and not living in poverty. We also created categorical variable scores of “parental interaction with their child” and “parental care for child’s physical care” (the four quantile groups) and performed multivariate linear regression analyses.

Fifth, we compared the effect sizes of the total number of “parental interaction with their child” and “parental care for child’s physical care” among adolescents living in poverty and not living in poverty.

Sixth, using the total sample, we also examined the impacts of poverty and parental involvement behaviors (i.e., the number of parental involvement behaviors, the number of parental interactions with their child, the amount of parental care for their child’s physical care) on adolescent’s self-esteem, and the interaction effect of poverty and parental involvement behaviors on adolescent’s self-esteem.

Seventh, a multivariate linear regression analysis was conducted to examine the association between each parental involvement behavior and self-esteem among adolescents in poverty. In this analysis, we also used the above categories of parental involvement and treated the missing data as a dummy variable. This analysis adjusted basic demographics and maternal childhood socioeconomic status in Model 1 and adjusted the caregiver’s psychological distress and relationship with their neighborhood in Model 2. Additionally, Model 3 included all parental involvement behaviors. Similar analyses were performed of adolescents not living in poverty.

All analyses were adjusted for response rates of each municipality (i.e., probability weight) and were conducted using STATA version 15.0 SE.

## 3. Results

### 3.1. Characteristics of the Participants in This Study

The mean and SD of adolescents’ self-esteem scores were 12.95 (SD = 6.09) among participants who were under the poverty line and 13.96 (SD = 5.96) among those who were not under the poverty line. Table 1 shows the distribution of characteristics among participants. Approximately 90% of the caregivers who responded to the questionnaire were mothers. Approximately 20% of the mothers were less than 40 years old, 20% of the caregivers were not married, 45% of adolescents had older sibling(s), and 50% had younger sibling(s). The caregivers living in poverty were more likely to be young and be unmarried than those not living in poverty. About 20% of the caregivers had experienced economic difficulties in their childhood and 40% of the mothers’ educational level was high school or less, in which these proportions were higher among the caregivers living in poverty than those not living in poverty. A total of 25% of the caregivers reported psychological distress. The caregivers living in poverty were more likely to report psychological distress than those not living in poverty. About 70% of the caregivers reported a poor relationship with their neighborhood. The differences in all characteristics other than child’s sex were statistically significant between the participants living in poverty and not living in poverty.

### 3.2. Distribution of Parental Involvement Behaviors

Table 2 shows the distribution of parental involvement behaviors among participants who were under the poverty line and those who were not under the poverty line. Approximately 30% of the caregivers helped their child study once or more a week (*p* = 0.175), talked about the news with their child almost every day (*p* < 0.001), played with their child as a physical activity once or more a month (*p* = 0.549), talked about TV shows with their child almost every day (*p* = 0.924), and talked about their child’s future frequently (*p* = 0.031), in which the participants not living in poverty were more likely to talk about the news with child and talk about child’s future than those living in poverty. About half of the caregivers talked about school life with their child almost every day (*p* < 0.001) and went out with their child once or more a week (*p* = 0.001), in which the participants not living in poverty were more likely to talk about school life with child and were less likely to go out with child than those living in poverty. Even though almost all caregivers hosted events for their children, this behavior was significantly less prominent in the participants living in poverty than those not living in poverty (*p* < 0.001). Parental care for their child’s physical health was different between participants living in poverty and not living in poverty (*p* < 0.001). Approximately 20% of the caregivers living in poverty reported the experience of not bringing a child to a doctor or hospital when they were sick, whereas this was only true for 10% of those not living in poverty. About 10% of the caregivers living in poverty did not have their child’s routine vaccinations or did not know, whereas this was only reported in 5% of those not living in poverty. Approximately 25% of the mothers living in poverty smoked in front of their children, compared to 10% of those not living in poverty. About 35% of the fathers smoked in front of their children, and 80% of the caregivers cooked for their children almost every day, in both groups of children.

### 3.3. Association between Parental Involvement Behaviors and Self-Esteem among Adolescents Living in Poverty and not Living in Poverty

Table 3 shows the results of the multiple linear regression analyses to examine the association between the total number of parental involvement behaviors and self-esteem among adolescents living in poverty and not living in poverty. Among adolescents living in poverty, a higher number of parental involvement behaviors was associated with higher self-esteem in the crude model (β = 0.42, 95% confidential interval (CI) = 0.33 to 0.50). In the adjusted models, the coefficient for the total number of parental involvement behaviors remained significant (Model 1: β = 0.29, 95% CI = 0.20 to 0.38; Model 2: β = 0.26, 95% CI = 0.17 to 0.35). Furthermore, we found the dose-response relationship between four quantile groups of parental involvement and self-esteem, in which the significant dose-response relationship remained in Model 2 (see Supplement Table S1). 

Among adolescents not living in poverty, it was also found the significant association between the total number of parental involvement behaviors and self-esteem (Crude model: β = 0.48, 95% CI = 0.42 to 0.53; Model1: β = 0.29, 95% CI = 0.23 to 0.35; Model2: β = 0.27, 95% CI = 0.20 to 0.33) (Table 3). A larger number of parental involvement behaviors were also associated with the dose-response relationship between four quantile groups of parental involvement and self-esteem (see Supplement Table S2). These results were similar to the results of adolescents living in poverty. 

### 3.4. Association between Parental Interaction with Child and Self-Esteem among Adolescents Living in Poverty and not Living in Poverty

Table 4 shows the results of the multiple linear regression analyses to examine the association between the total number of parental interactions with their child and self-esteem among adolescents living in poverty and not living in poverty. We found that a high amount of parental interaction with a child was found to be associated with high self-esteem even while adjusting for covariates, which was similar to the results in both adolescents living in poverty (Crude model: β = 0.47, 95% CI = 0.37 to 0.57; Model 1: β = 0.33, 95% CI = 0.22 to 0.44; β = 0.30, 95% CI = 0.19 to 0.40) and not living in poverty (Crude model: β = 0.57, 95% CI = 0.50 to 0.63; Model 1: β = 0.34, 95% CI = 0.26 to 0.41; β = 0.31, 95% CI = 0.24 to 0.38). 

Additionally, four quantile groups of parental interaction with a child had a dose-response relationship with self-esteem among adolescents living in poverty (see Supplement Table S1). Compared to Q1, Q4 showed a higher score of self-esteem in the crude model (β = 2.40, 95% CI = 1.83 to 2.97). Similarly, Q4 showed a higher score of self-esteem than Q1 among adolescents not living in poverty (β = 3.03, 95% CI = 2.64 to 3.42) (see Supplement Table S2).

### 3.5. Association between Parental Care for Child’s Physical Health and Self-Esteem among Adolescents Living in Poverty and not Living in Poverty

Table 5 shows the results of the multiple linear regression analyses to examine the association between the amount of parental care for a child’s physical health and self-esteem among adolescents living in poverty and not living in poverty. Among adolescents living in poverty, the results showed that a high amount of parental care for a child’s physical health was associated with high self-esteem even when there was an adjustment for confounders (Crude model: β = 0.41, 95% CI = 0.22 to 0.60; Model 1: β = 0.23, 95% CI = 0.03 to 0.42). However, in Model 2, the coefficient was not significant (β = 0.18, 95% CI = −0.02 to 0.38). Among adolescents not living in poverty, the results showed that a high amount of parental care for a child’s physical health was associated with high self-esteem, in which the significant relationship remained in Model 2 (β = 0.20, 95% CI = 0.05 to 0.35).

In terms of the association between four quantile groups of parental care for child’s physical health and self-esteem, Q4 showed a higher score for self-esteem in the crude model compared to Q1 (β = 0.88, 95% CI = 0.31 to 1.46) among adolescents living in poverty (see Supplement Table S1). Among adolescents not living in poverty, Q4 showed a higher score of self-esteem than Q1, in which the significant relationship remained in Model 2 (β = 0.52, 95% CI = 0.15 to 0.90) (see Supplement Table S2).

### 3.6. Comparison among Effect Size of Parental Interaction with Child and Parental Care for Child’s Physical Health

Among adolescents living in poverty, the results of the analysis included both parental interaction with their child and parental care for the child’s physical health, which were adjusted for all covariates, and showed that parental interaction with the child had a larger effect size (β = 0.29, 95% CI = 0.19 to 0.40) than that of parental care for child’s physical health (β = 0.15, 95% CI = −0.05 to 0.34). However, the differences between the coefficients were not significant (*p* = 0.213).

Among adolescents not living in poverty, the results of the analysis included both parental interaction with their child and parental care for the child’s physical health, which were adjusted for all covariates, and showed that parental interaction with the child had a larger effect size (β = 0.31, 95% CI = 0.24 to 0.38) than that of parental care for child’s physical health (β = 0.20, 95% CI = 0.05 to 0.35), in which the differences between the coefficients were significant (*p* = 0.044). 

### 3.7. Interaction between Poverty and Paretal Involvement Behaviors 

Using the total sample, poverty and parental involvement behaviors (i.e., the total number of “parental involvement behaviors”, the total number of “parental interaction with their child”, and the total amount of “parental care for child’s physical health”) were associated with self-esteem even though all covariates were adjusted (see Supplement Table S3). Furthermore, in terms of the interaction between poverty and parental involvement behaviors, we did not find any statistically significant interaction effects (parental involvement behaviors: *p* = 0.311; parental interactions with their child: *p* = 0.258; parental care for their child’s physical health: *p* = 0.706).

### 3.8. Association between Each Parental Involvement Behaviors and Self-Esteem among Adolescents Living in Poverty and not Living in Poverty

We also examined the association between each parental involvement behavior and self-esteem among adolescents living in poverty (see Supplement Table S4). In Model 2, which adjusted basic demographics, maternal childhood socioeconomic status, caregiver’s psychological distress, and relationship with their neighborhood, parental involvement behaviors were significantly associated with self-esteem: talking about school life, talking about news, playing with their child, talking about TV shows, talking about the child’s future, hosting events for their child, and cooking for their child. Model 3 included all parental involvement behaviors and coefficients: talking about news (β = 0.58, 95% CI = 0.07 to 1.10), playing with their child (β = 0.80, 95% CI = 0.28 to 1.32), talking about TV shows (β = 0.82, 95% CI = 0.28 to 1.32), talking about the child’s future (β = 1.20, 95% CI = 0.69 to 1.70), and hosting events for their child (β = 1.24, 95% CI = 0.03 to 2.45), which showed a significant positive association with self-esteem.

Among adolescents not living in poverty (see Supplement Table S5), parental involvement behaviors were significantly associated with self-esteem: talking about school life, talking about news, playing with their child, talking about TV shows, going out with child, talking about their child’s future, and having no experience of not bringing the child to a doctor when they were sick, in Model 2. Coefficients in Model 3: talking about school life (β = 0.98, 95% CI = 0.64 to 1.32), talking about news (β = 0.37, 95% CI = 0.03 to 0.71), playing with their child (β = 0.59, 95% CI = 0.24 to 0.95), talking about their child’s future (β = 0.97, 95% CI = 0.64 to 1.30), and having no experience of not bringing the child to a doctor when they were sick (β = 0.76, 95% CI = 0.29 to 1.22), showed a significant positive association with self-esteem.

## 4. Discussion

In this study, we found that large numbers of parental involvement behaviors were associated with a high self-esteem score of adolescents living in poverty. We examined two aspects of parental involvement behavior—parental interaction with their child and parental care for the child’s physical health—and found that parental interaction with their child showed a strong association with the child’s self-esteem. These associations also exist in adolescents not living in poverty. 

Our findings indicate that the number of parental involvement behaviors could be an indicator of self-esteem in adolescents living in poverty, which is consistent with previous studies showing that the quality of parenting is a mediator of the association between child poverty and self-esteem [34,35,36]. Furthermore, the results of this study can be explained by the Family Stress Model (FSM) which provides that family financial problems affect psychological adjustment including self-esteem in adolescents via parental emotional distress [55]. As the FSM indicates, parental psychological distress was also associated with child’s self-esteem in the adjusted model. Our findings provide evidence of the benefits of intervening to encourage caregivers living in poverty to engage in parental involvement behaviors. In particular, the parental involvement behaviors which are important to increase were talking about news, playing with the child, talking about the child’s future, and hosting events for the child. Furthermore, the assessment of the quantity of parenting involvement could be used to evaluate the effect of a public health approach related to parenting, especially when the assessment of the quality is difficult.

Moreover, we have found that parental care for the child’s physical health showed no significant association with self-esteem in adolescents living in poverty in the adjusted model, whereas the association was significant in adolescents not living in poverty. Parental involvement behaviors are shaped by a parenting style which is defined as parents’ attitudes, beliefs, and behavior, which are all associated with the offspring’s self-esteem [56]. Parenting style includes authoritative, authoritarian, permissive, and rejecting-neglectful [57], which are characterized by demandingness (control) and responsiveness (warmth). Generally, an authoritative style, which combines high demandingness with responsiveness, consistently leads to offspring’s positive outcomes such as mental health [58,59,60]. An authoritative style in this study might reflect a higher number of parental interactions, which is why parental interaction with their child was strongly associated with high self-esteem among adolescents. Conversely, other studies show that an authoritarian style, which combines high demandingness with low responsiveness, is associated with child health-related behaviors, such as feeding and sedentary behavior [61,62]. According to the results of these previous studies, parental care for the child’s physical health might be reflected in parenting with higher control. Therefore, parental care for their child’s physical health was not associated with self-esteem among families living in poverty, although we should interpret our findings carefully—especially because having experience of not visiting the hospital for child might be affected by the access to the hospital which differ from rural and urban area. 

We also found the impacts of specific parenting involvement behaviors on adolescent self-esteem which may contribute to developing a public health approach. For example, there is a growing body of literature related to brief text-based intervention via mobile phones which can approach many people living in poverty [63]. Previous studies indicate that the target behavior is episodic rather than habitual or ongoing [64], and users prefer goal-directed messaging [65]. Based on these recommendations, it may be helpful to provide brief, concrete, goal-directed parental involvement behaviors for caregivers. For example, recommending caretakers “talk with your child about their future once a month”, “host events such as birthday parties for your child”, or “cook for your child as often as much as possible” may be more effective than vague messages, such as “increase interaction with your child.” According to the FSM, the caregivers living in poverty who need to improve their parental involvement behaviors may be more likely to have mental health issues. Therefore, providing the brief, concrete, goal-directed parental involvement behaviors may also helpful for these caregivers because they may not afford to consider their parental involvement behaviors. Further studies are needed to examine the efficacy of interventions that use brief, concrete, goal-directed messages to increase specific parental involvement behaviors to improve the child’s self-esteem. Moreover, we should examine the efficacy of interventions among family living in poverty, because the findings of this study showed that caregivers living in poverty were less likely to engage almost all parenting involvement behaviors than those not living in poverty.

The current study has several limitations. First, a reverse association between parental involvement behaviors and a child’s self-esteem is possible due to the cross-sectional design of the study. In other words, it might be easier for a parent to interact with a child with high self-esteem than with low self-esteem. To explore the causal association, further randomized controlled trials and/or longitudinal studies are needed. Second, our findings might differ between countries, because the parental involvement behaviors of this study were selected to suit the Japanese context. For instance, “cooking for their child” is one key parental involvement behaviors for a child’s physical health. Tani et al. [66] have found the association between home cooking and child obesity, using a Japanese sample. However, there are some cultures in which the family goes out to eat daily. Thus, one must be mindful when applying our findings to other countries. Third, this study was conducted in only one rural prefecture in Japan. Our findings would be enhanced by using a representative sample of the Japanese population with better response rates. Fourth, we merely assessed the quantity of parental involvement behaviors, not the quality. The caregivers who engaged in a large number of parental involvement behaviors might also engage in high-quality parenting practices, whereas we did not measure this in our study. Further studies to examine both the quantity and quality of parental involvement behaviors are needed. Fifth, we did not use the established measurement of parental involvement behaviors, as the Cronbach’s alpha of this measurement was poor. It is important to discuss which parental involvement behaviors should be included in the measurement.

## 5. Conclusions

In conclusion, our findings suggest that adolescents have high self-esteem when their caregivers engage in parental involvement behaviors, even though the adolescents live in poverty. To empower adolescents in poverty, caregivers must provide both parental interactions with their child and parental care for their child’s physical health.

## Figures and Tables

**Table 1:** Descriptive characteristics of the participants in this study (*n* = 10,784).

Characteristics		Total (*n* = 10,784)		Not Living in Poverty (*n* = 7088; 65.7%)		Living in Poverty (*n* = 3696; 34.3%)		
		*n*	% or Mean (SD)	*n*	% or Mean (SD)	*n*	% or Mean (SD)	*p* for *t*-Test or Chi-Squared Test
Basic demographics								
Respondent	Mother	9384	87.0	6153	86.8	3231	87.4	0.005
	Father	1130	10.5	775	10.9	355	9.6	
	Grandparent	110	1.0	58	0.8	52	1.4	
	Others	60	0.6	34	0.5	26	0.7	
	Missing	100	0.9	68	1.0	32	0.9	
Child sex	Male	5025	46.6	3317	46.8	1708	46.2	0.675
	Female	5525	51.2	3613	51	1912	51.7	
	Missing	234	2.2	158	2.2	76	2.1	
Grade	5th	3117	28.9	1955	27.6	1162	31.4	<0.001
	8th	3498	32.4	2339	33	1159	31.4	
	11th	4169	38.7	2794	39.4	1375	37.2	
Maternal age	<40 years	1837	17.0	994	14.0	843	22.8	<0.001
	40 years–<50 years	6841	63.4	4689	66.2	2152	58.2	
	50 years+	1344	12.5	971	13.7	373	10.1	
	Missing	762	7.1	434	6.1	328	8.9	
Marital status	Married	8696	80.6	6213	87.7	2483	67.2	<0.001
	Unmarried/divorced/widowed	1974	18.3	802	11.3	1172	31.7	
	Missing	114	1.1	73	1.0	41	1.1	
Having older sibling	No	6175	57.3	4121	58.1	2054	55.6	0.011
	Yes	4609	42.7	2967	41.9	1642	44.4	
Having younger sibling	No	5527	51.3	3716	52.4	1811	49.0	0.001
	Yes	5257	48.7	3372	47.6	1885	51.0	
Caregiver’s childhood socioeconomic status								
Economic difficulties in childhood	No	8739	81.0	6023	85.0	2716	73.5	<0.001
	Yes	2045	19.0	1065	15.0	980	26.5	
Maternal education	High school or less	3944	36.6	2111	29.8	1833	49.6	<0.001
	Some college	4654	43.2	3331	47.0	1323	35.8	
	College or more	1448	13.4	1207	17.0	241	6.5	
	Other/Unknown	53	0.5	31	0.4	22	0.6	
	Missing	685	6.4	408	5.8	277	7.5	
Caregiver’s other possible confounders								
Psychological distress (K6)	0–<5	7441	69.0	5344	75.4	2097	56.7	
	5–<13	2442	22.6	1323	18.7	1119	30.3	<0.001
	13+	526	4.9	192	2.7	334	9.0	
	Missing	375	3.5	229	3.2	146	4.0	
Relationship with neighborhood	High	3109	28.8	2144	30.2	965	26.1	<0.001
	Low	7153	66.3	4606	65.0	2547	68.9	
	Missing	522	4.8	338	4.8	184	5.0	

**Table 2:** Parental involvement behaviors among adolescents living in poverty and not living in poverty (*n* = 10,784).

Parental Involvement Behaviors		Not Living in Poverty (*n* = 7088; 65.7%)		Living in Poverty (*n* = 3696; 34.3%)		*p* for Chi-Squared Test
		*n*	%	*n*	%	
Parental interaction with child						
Helping child study	<1 a week	4775	67.4	2424	65.6	0.175
	1+ a week	2039	28.8	1120	30.3	
	Missing	274	3.9	152	4.1	
Talking about school life	<5 a week	3098	43.7	1812	49.0	<0.001
	Almost everyday	3728	52.6	1746	47.2	
	Missing	262	3.7	138	3.7	
Talking about news	<3 a week	4648	65.6	2634	71.3	<0.001
	3+ a week	2156	30.4	916	24.8	
	Missing	284	4.0	146	3.9	
Playing with child (physical activity)	Rarely	4754	67.1	2500	67.6	0.549
	1+ a month	2043	28.8	1045	28.3	
	Missing	291	4.1	151	4.1	
Talking about TV shows with child	<5 a week	4281	60.4	2232	60.4	0.924
	Almost everyday	2538	35.8	1320	35.7	
	Missing	269	3.8	144	3.9	
Cooking with child	<3 a month	5526	78.0	2744	74.2	<0.001
	1+ a week	1279	18.0	808	21.9	
	Missing	283	4.0	144	3.9	
Going out with child	<3 a month	3259	45.9	1578	42.7	0.001
	1+ a week	3577	50.5	1987	53.8	
	Missing	252	3.6	131	3.5	
Talking about child’s future	Sometimes or less	4870	68.7	2630	71.2	0.031
	Frequently	1989	28.1	959	25.9	
	Missing	229	3.2	107	2.9	
Hosting events for child	No	41	0.6	103	2.8	<0.001
	Yes	6844	96.6	3499	94.7	
	Missing	203	2.9	94	2.5	
Parental care for child physical health						
Having experience of not visiting the hospital for child	Yes	743	10.5	670	18.1	<0.001
	No	6034	85.1	2860	77.4	
	Missing	311	4.4	166	4.5	
History of routine vaccination	No/unknown	301	4.3	332	9.0	
	Yes	6498	91.7	3209	86.8	<0.001
	Missing	289	4.1	155	4.2	
Maternal smoking in front of child	Smoking in front of child	788	11.1	955	25.9	<0.001
	Never smoking/smoking but not in front of child	5937	83.8	2466	66.7	
	Missing	363	5.1	275	7.4	
Paternal smoking in front of child	Smoking in front of child	2333	32.9	1383	37.4	<0.001
	Never smoking/smoking but not in front of child	4004	56.5	1256	34.0	
	Missing	751	10.6	1057	28.6	
Cooking for child	<6 (days) week	985	13.9	707	19.1	<0.001
	Almost everyday	5858	82.6	2867	77.6	
	Missing	245	3.5	122	3.3	

**Table 3:** The association between the total number of parental involvement behaviors and self-esteem among adolescents living in poverty (*n* = 3696) and not living in poverty (*n* = 7088).

		Living in Poverty			Not Living in Poverty		
		Crude	Model 1 ^a^	Model 2 ^b^	Crude	Model 1 ^a^	Model 2 ^b^
		β (95% CI)	β (95% CI)	β (95% CI)	β (95% CI)	β (95% CI)	β (95% CI)
Parental involvement behaviors	Total score (0–14)	0.42 ** (0.33 to 0.50)	0.29 ** (0.20 to 0.38)	0.26 ** (0.17 to 0.35)	0.48 ** (0.42 to 0.53)	0.29 ** (0.23 to 0.35)	0.27 ** (0.20 to 0.33)
Respondent	Mother		Ref	Ref		Ref	Ref
	Father		0.85 ** (0.07 to 1.62)	0.71 (−0.05 to 1.48)		−0.12 (−0.58 to 0.34)	−0.22 (−0.68 to 0.24)
	Grandparent		0.56 (−1.13 to 2.24)	0.28 (−1.38 to 1.95)		−1.41 (−3.02 to 0.20)	−1.74 ** (−3.36 to −0.11)
	Others		−0.06 (−2.88 to 2.76)	−0.15 (−3.05 to 2.75)		−0.38 (−2.46 to 1.71)	−0.48 (−2.51 to 1.55)
Child sex	Male		Ref	Ref		Ref	Ref
	Female		−1.37 ** (−1.76 to −0.96)	−1.38 ** (−1.78 to −0.97)		−1.39 ** (−1.67 to −1.12)	−1.38 ** (−1.66 to −1.10)
Grade	5th		Ref	Ref		Ref	Ref
	8th		−2.40 ** (−2.95 to −1.85)	−2.40 ** (−2.95 to −1.85)		−2.60 ** (−2.98 to −2.23)	−2.61 ** (−2.99 to −2.24)
	11th		−2.67 ** (−3.25 to −2.10)	−2.69 ** (−3.27 to −2.12)		−3.20 ** (−3.26 to −2.79)	−3.26 ** (−3.66 to −2.85)
Maternal age	<40 years		Ref	Ref		Ref	Ref
	40 years–<50 years		0.32 (−0.22 to 0.87)	0.23 (−0.31 to 0.76)		−0.05 (−0.48 to 0.37)	−0.08 (−0.51 to 0.35)
	50 years+		−0.16 (−0.99 to 0.67)	−0.24 (−1.06 to 0.59)		−0.44 (−1.02 to 0.14)	−0.46 (−1.04 to 0.12)
Marital status	Married		Ref	Ref		Ref	Ref
	Unmarried/divorced/widowed		0.34 (−0.13 to 0.81)	0.35 (−0.11 to 0.82)		0.25 (−0.24 to 0.73)	0.25 (−0.23 to 0.75)
Having older sibling	No		Ref	Ref		Ref	Ref
	Yes		0.04 (−0.41 to 0.48)	−0.05 (−0.49 to 0.39)		−0.29 (−0.59 to 0.02)	−0.32 * (−0.63 to −0.02)
Having younger sibling	No		Ref	Ref		Ref	Ref
	Yes		0.24 (−0.21 to 0.69)	0.16 (−0.28 to 0.27)		−0.28 (−0.59 to 0.04)	−0.33 * (−0.65 to −0.02)
Economic difficulties in childhood	No		Ref	Ref		Ref	Ref
	Yes		−0.37 (−0.84 to 0.09)	−0.19 (−0.65 to 0.27)		−0.31 (−0.70 to 0.08)	−0.21 (−0.60 to 0.18)
Maternal education	High school or less		Ref	Ref		Ref	Ref
	Some college		0.33 (−0.12 to 0.78)	0.33 (−0.12 to 0.77)		0.33 * (0.003 to 0.65)	0.33 * (0.01 to 0.65)
	College or more		0.78 (−0.09 to 1.66)	0.71 (−0.17 to 1.59)		0.63 ** (0.19 to 1.07)	0.66 ** (0.22 to 1.09)
	Other/Unknown		0.47 (−2.15 to 3.08)	0.55 (−2.06 to 3.15)		0.12 (−2.07 to 2.32)	0.23 (−1.98 to 2.43)
Caregiver’s psychological distress (K6)	0–<5			Ref			Ref
	5–<13			0.56 (−0.22 to 1.33)			0.81 (−0.17 to 1.80)
	13+			1.19 ** (0.44 to 1.94)			1.51 ** (0.57 to 2.46)
Caregiver’s relationship with neighborhood	High			Ref			Ref
	Low			0.99 ** (0.52 to 1.46)			0.50 ** (0.20 to 0.81)
R^2^		0.029	0.087	0.100	0.040	0.116	0.122

^a^ Adjusted for child sex, grade, respondent, maternal age, marital status, having old sibling, having young sibling, maternal economic difficulties in childhood, maternal education, and all 34 municipalities in Kochi prefecture (The coefficients were not shown). ^b^ Adjusted for caregiver’s psychological distress and relationship with neighborhood. ** *p* < 0.01, * *p* < 0.05.

**Table 4:** The association between total number of parental interactions with child and self-esteem among adolescents living in poverty (*n* = 3696) and not living in poverty (*n* = 7088).

		Living in Poverty			Not Living in Poverty		
		Crude	Model 1 ^a^	Model 2 ^b^	Crude	Model 1 ^a^	Model 2 ^b^
		β (95% CI)	β (95% CI)	β (95% CI)	β (95% CI)	β (95% CI)	β (95% CI)
Parental interaction with child	Total score (0–9)	0.47 ** (0.37 to 0.57)	0.33 ** (0.22 to 0.44)	0.30 ** (0.19 to 0.40)	0.57 ** (0.50 to 0.63)	0.34 ** (0.26 to 0.41)	0.31 ** (0.24 to 0.38)
Respondent	Mother		Ref	Ref		Ref	Ref
	Father		0.87 * (0.09 to 1.65)	0.71 (−0.05 to 1.48)		−0.12 (−0.58 to 0.34)	−0.22 (−0.68 to 0.24)
	Grandparent		0.61 (−1.09 to 2.31)	0.28 (−1.38 to 1.95)		−1.45 (−3.05 to 0.16)	−1.76 ** (−3.38 to −0.14)
	Others		−0.33 (−3.13 to 2.48)	−0.15 (−3.05 to 2.75)		−0.40 (−2.47 to 1.67)	−0.50 (−2.52 to 1.52)
Child sex	Male		Ref	Ref		Ref	Ref
	Female		−1.40 ** (−1.81 to −1.00)	−1.38 ** (−1.78 to −0.97)		−1.42 ** (−1.70 to −1.14)	−1.41 ** (−1.68 to −1.13)
Grade	5th		Ref	Ref		Ref	Ref
	8th		−2.39 ** (−2.94 to −1.84)	−2.40 ** (−2.95 to −1.85)		−2.56 ** (−2.93 to −2.18)	−2.56 ** (−2.94 to −2.19)
	11th		−2.69 ** (−3.27 to −2.12)	−2.69 ** (−3.27 to −2.12)		−3.18 ** (−3.59 to −2.78)	−3.23 ** (−3.64 to −2.83)
Maternal age	<40 years		Ref	Ref		Ref	Ref
	40 years–<50 years		0.38 (−0.16 to 0.92)	0.23 (−0.31 to 0.76)		0.02 (−0.41 to 0.44)	−0.02 (−0.44 to 0.41)
	50 years+		−0.04 (−0.88 to 0.79)	−0.24 (−1.06 to 0.59)		−0.33 (−0.91 to 0.25)	−0.35 (−0.94 to 0.23)
Marital status	Married		Ref	Ref		Ref	Ref
	Unmarried/divorced/widowed		0.15 (−0.32 to 0.61)	0.35 (−0.11 to 0.82)		0.02 (−0.46 to 0.50)	0.05 (−0.43 to 0.53)
Having older sibling	No		Ref	Ref		Ref	Ref
	Yes		0.03 (−0.42 to 0.47)	−0.05 (−0.49 to 0.39)		−0.27 (−0.58 to 0.03)	−0.31 * (−0.61 to −0.005)
Having younger sibling	No		Ref	Ref		Ref	Ref
	Yes		0.26 (−0.19 to 0.71)	0.16 (−0.28 to 0.27)		−0.24 (−0.55 to 0.07)	−0.30 (−0.61 to 0.01)
Economic difficulties in childhood	No		Ref	Ref		Ref	Ref
	Yes		−0.42 (−0.88 to 0.04)	−0.19 (−0.65 to 0.27)		−0.32 (−0.71 to 0.07)	−0.22 (−0.61 to 0.17)
Maternal education	High school or less		Ref	Ref		Ref	Ref
	Some college		0.41 (−0.04 to 0.86)	0.33 (−0.12 to 0.77)		0.38 * (0.06 to 0.70)	0.38 * (0.05 to 0.70)
	College or more		0.92 * (0.04 to 1.79)	0.71 (−0.17 to 1.59)		0.72 ** (0.28 to 1.15)	0.73 ** (0.30 to 1.17)
	Other/Unknown		0.43 (−2.15 to 3.02)	0.55 (−2.06 to 3.15)		0.08 (−2.12 to 2.29)	0.19 (−2.03 to 2.41)
Caregiver’s psychological distress (K6)	0–<5			Ref			Ref
	5–<13			0.56 (−0.22 to 1.33)			0.82 (−0.16 to 1.80)
	13+			1.19 ** (0.44 to 1.94)			1.55 ** (0.61 to 2.49)
Caregiver’s relationship with neighborhood	High			Ref			Ref
	Low			0.99 ** (0.52 to 1.46)			0.49 ** (0.19 to 0.80)
R^2^		0.026	0.087	0.100	0.042	0.116	0.122

^a^ Adjusted for child sex, grade, respondent, maternal age, marital status, having old sibling, having young sibling, maternal economic difficulties in childhood, maternal education, and all 34 municipalities in Kochi prefecture (The coefficients were not shown). ^b^ Adjusted for caregiver’s psychological distress and relationship with neighborhood. ** *p* < 0.01, * *p* < 0.05.

**Table 5:** The association between the amount of parental care for a child’s physical health and self-esteem among adolescents living in poverty (*n* = 3696) and not living in poverty (*n* = 7088).

		Living in Poverty			Not Living in Poverty		
		Crude	Model 1 ^a^	Model 2 ^b^	Crude	Model 1 ^a^	Model 2 ^b^
		β (95% CI)	β (95% CI)	β (95% CI)	β (95% CI)	β (95% CI)	β (95% CI)
Parental care for child’s physical health	Total score (0–5)	0.41 ** (0.22 to 0.60)	0.23 * (0.03 to 0.42)	0.18 (−0.02 to 0.38)	0.40 ** (0.26 to 0.54)	0.24 * (0.09 to 0.39)	0.20 * (0.05 to 0.35)
Respondent	Mother		Ref	Ref		Ref	Ref
	Father		0.70 (−0.08 to 1.48)	0.57 (−0.19 to 1.34)		−0.27 (−0.73 to 0.19)	−0.37 (−0.83 to 0.10)
	Grandparent		0.21 (−1.51 to 1.93)	−0.05 (−1.75 to 1.65)		−1.54 (−3.19 to 0.10)	−1.93 * (−3.58 to −0.28)
	Others		−0.18 (−2.99 to 2.63)	−0.27 (−3.16 to 2.63)		−0.39 (−2.40 to 1.63)	−0.51 (−2.46 to 1.45)
Child sex	Male		Ref	Ref		Ref	Ref
	Female		−1.20 ** (−1.61 to −0.79)	−1.23 ** (−1.63 to −0.83)		−1.23 ** (−1.51 to −0.95)	−1.23 ** (−1.51 to −0.95)
Grade	5th		Ref	Ref		Ref	Ref
	8th		−2.71 ** (−3.25 to −2.17)	−2.67 ** (−3.21 to −2.14)		−3.00 ** (−3.37 to −2.63)	−2.97 ** (−3.34 to −2.60)
	11th		−3.21 ** (−3.75 to −2.66)	−3.16 ** (−3.70 to −2.62)		−3.80 ** (−4.19 to −3.42)	−3.81 ** (−4.20 to −3.43)
Maternal age	<40 years		Ref	Ref		Ref	Ref
	40 years–<50 years		0.29 (−0.25 to 0.83)	0.19 (−0.35 to 0.73)		−0.08 (−0.51 to 0.35)	−0.10 (−0.53 to 0.33)
	50 years+		−0.27 (−1.11 to 0.57)	−0.33 (−1.17 to 0.50)		−0.53 (−1.12 to 0.06)	−0.53 (−1.13 to 0.06)
Marital status	Married		Ref	Ref		Ref	Ref
	Unmarried/divorced/widowed		0.29 (−0.20 to 0.77)	0.30 (−0.19 to 0.78)		0.13 (−0.36 to 0.63)	0.13 (−0.36 to 0.62)
Having older sibling	No		Ref	Ref		Ref	Ref
	Yes		−0.02 (−0.46 to 0.43)	−0.10 (−0.54 to 0.34)		−0.35 * (−0.65 to −0.04)	−0.38 * (−0.68 to −0.07)
Having younger sibling	No		Ref	Ref		Ref	Ref
	Yes		0.22 (−0.23 to 0.68)	0.15 (−0.30 to 0.60)		−0.28 (−0.60 to 0.03)	−0.34 * (−0.66 to −0.03)
Economic difficulties in childhood	No		Ref	Ref		Ref	Ref
	Yes		−0.39 (−0.85 to 0.08)	−0.19 (−0.66 to 0.27)		−0.32 (−0.71 to 0.08)	−0.21 (−0.61 to 0.18)
Maternal education	High school or less		Ref	Ref		Ref	Ref
	Some college		0.37 (−0.09 to 0.83)	0.36 (−0.09 to 0.81)		0.38 * (0.06 to 0.70)	0.38 * (0.06 to 0.70)
	College or more		0.83 (−0.04 to 1.71)	0.75 (−0.13 to 1.63)		0.71 ** (0.27 to 1.16)	0.74 ** (0.30 to 1.18)
	Other/Unknown		0.50 (−2.17 to 3.18)	0.58 (−2.07 to 3.23)		0.24 (−1.99 to 2.46)	0.35 (−1.88 to 2.59)
Caregiver’s psychological distress (K6)	0–<5			Ref			Ref
	5–<13			0.59 (−0.18 to 1.37)			0.81 (−0.17 to 1.80)
	13+			1.24 * (0.50 to 2.00)			1.55 ** (0.61 to 2.50)
Caregiver’s relationship with neighborhood	High			Ref			Ref
	Low			1.12 ** (0.65 to 1.59)			0.65 ** (0.34 to 0.95)
R^2^		0.006	0.077	0.092	0.005	0.106	0.113

^a^ Adjusted for child sex, grade, respondent, maternal age, marital status, having old sibling, having young sibling, maternal economic difficulties in childhood, maternal education, and all 34 municipalities in Kochi prefecture (The coefficients were not shown). ^b^ Adjusted for caregiver’s psychological distress and relationship with neighborhood. ** *p* < 0.01, * *p* < 0.05.

## Supplementary Materials

The following are available online at https://www.mdpi.com/1660-4601/17/17/6277/s1, Table S1: The associations of parental involvement behaviors, parental interaction with child, and parental care for child’s physical health with self-esteem among adolescents living in poverty (*n* = 3696). Table S2. The association between total number of parental involvement behaviors and self-esteem among adolescents not living in poverty (*n* = 7088). Table S3. The associations of poverty and parental involvement behaviors with self-esteem in total sample (*n* = 10,784). Table S4. Association between parental involvement behaviors and young adolescent’s self-esteem among adolescents living in poverty (*n* = 3696). Table S5. Association between parental involvement behaviors and young adolescent’s self-esteem among adolescents living in poverty (*n* = 7088).
