# Supplementary material for: The Association between Parental Involvement Behavior and Self-Esteem among Adolescents Living in Poverty: Results from the K-CHILD Study
Source: Int J Environ Res Public Health. 2020 Aug 28;17(17):6277. doi: 10.3390/ijerph17176277 (PMC7504259; doi:10.3390/ijerph17176277)
Supplement: Supplementary file 1 [file ijerph-17-06277-s001.zip › ijerph-895855-supplementary.docx]

| **Table S1.** The associations of parental involvement behaviors, parental interaction with child, and parental care for child's physical health with self-esteem among adolescents living in poverty (n=3,696). | | | | |
| --- | --- | --- | --- | --- |
|  |  | **Crude** | **Model 1^a^** | Model 2^b^ |
|  |  | β (95%CI) | β (95%CI) | β (95%CI) |
| Parental involvement behaviors |  |  |  |  |
| Quartile | Q1 (n=1,052, 28.5%) | Ref | Ref | Ref |
|  | Q2 (n=1,081, 29.2%) | 0.43 (−0.10 to 0.96) | 0.20 (−0.33 to 0.74) | 0.13 (−0.40 to 0.67) |
|  | Q3 (n=918, 24.8%) | 1.88^**^ (1.32 to 2.45) | 1.28^**^ (0.69 to 1.87) | 1.11^**^ (0.52 to 1.70) |
|  | Q4 (n=645, 17.4%) | 2.66^**^ (2.04 to 3.29) | 1.77^**^ (1.09 to 2.45) | 1.58^**^ (0.90 to 2.27) |
| Parental interaction with child |  |  |  |  |
| Quartile | Q1 (n=1,257, 34.0%) | Ref | Ref | Ref |
|  | Q2 (n=614, 16.6%) | 0.37 (−0.23 to 0.98) | 0.15 (−0.45 to 0.76) | 0.12 (−0.48 to 0.72) |
|  | Q3 (n=1,074, 29.1%) | 1.64^**^ (1.11 to 2.16) | 1.01^**^ (0.47 to 1.55) | 0.91^**^ (0.37 to 1.44) |
|  | Q4 (n=751, 20.3%) | 2.40^**^ (1.83 to 2.97) | 1.67^**^ (1.04 to 2.29) | 1.51^**^ (0.89 to 2.13) |
| Parental care for child's physical health |  |  |  |  |
| Quartile | Q1 (n=1,861, 50.3%) | Ref | Ref | Ref |
|  | Q3 (n=1,156, 31.3%) | 0.26 (−0.21 to 0.73) | 0.003 (−0.48 to 0.48) | −0.05 (−0.53 to 0.42) |
|  | Q4 (n=679, 18.4%) | 0.88^*^ (0.31 to 1.46) | 0.37 (−0.24 to 0.98) | 0.27 (−0.33 to 0.88) |
| ^a^ Adjusted for child sex, grade, respondent, maternal age, marital status, having old sibling, having young sibling, maternal economic difficulties in childhood, maternal education, and all 34 municipalities in Kochi prefecture.  ^b^ Adjusted for caregiver's psychological distress and relationship with neighborhood. ** p<0.001, * p<0.005. | | | | |
|  |  |  |  |  |
|  |  |  |  |  |

| **Table S2.** The association between total number of parental involvement behaviors and self-esteem among adolescents not living in poverty (n=7,088). | | | | |
| --- | --- | --- | --- | --- |
|  |  | **Crude** | **Model 1^a^** | Model 2^b^ |
|  |  | β (95%CI) | β (95%CI) | β (95%CI) |
| Parental involvement behaviors |  |  |  |  |
| Quartile | Q1 (n=2.399, 33.8%) | Ref | Ref | Ref |
|  | Q2 (n=1,991, 28.1%) | 1.25^**^ (0.90 to 1.61) | 0.79^**^ (0.44 to 1.15) | 0.72^**^ (0.37 to 1.08) |
|  | Q3 (n=1,692, 23.9%) | 2.18^**^ (1.80 to 2.56) | 1.36^**^ (0.97 to 1.76) | 1.25^**^ (0.86 to 1.65) |
|  | Q4 (n=1,006, 14.2%) | 3.57^**^ (3.11 to 4.02) | 2.18^**^ (1.70 to 2.66) | 2.02^**^ (1.54 to 2.51) |
| Parental interaction with child |  |  |  |  |
| Quartile | Q1 (n=2,425, 34.2%) | Ref | Ref | Ref |
|  | Q2 (n=2,158, 30.5%) | 0.94^**^ (0.59 to 1.28) | 0.37^*^ (0.03 to 0.71) | 0.32 (−0.02 to 0.67) |
|  | Q3 (n=971, 13.7%) | 2.00^**^ (1.55 to 2.45) | 1.11^**^ (0.66 to 1.56) | 1.00^**^ (0.55 to 1.45) |
|  | Q4 (n=1,534, 21.6%) | 3.03^**^ (2.64 to 3.42) | 1.69^**^ (1.27 to 2.11) | 1.57^**^ (1.14 to 1.99) |
| Parental care for child physical health |  |  |  |  |
| Quartile | Q1 (n=1,964, 27.7%) | Ref | Ref | Ref |
|  | Q3 (n=2,433, 34.3%) | 0.31 (−0.06 to 0.69) | 0.10 (−0.26 to 0.46) | 0.05 (−0.32 to 0.41) |
|  | Q4 (n=2,691, 38.0%) | 0.95^**^ (0.59 to 1.31) | 0.63^**^ (0.25 to 1.00) | 0.52^**^ (0.15 to 0.90) |
| ^a^ Adjusted for child sex, grade, respondent, maternal age, marital status, having old sibling, having young sibling, maternal economic difficulties in childhood, maternal education, and all 34 municipalities in Kochi prefecture.  ^b^ Adjusted for caregiver's psychological distress and relationship with neighborhood. ** p<0.01, * p<0.05. | | | | |
|  |  |  |  |  |
|  |  |  |  |  |

| **Table S3.** The associations of poverty and parental involvement behaviors with self-esteem in total sample (n=10,784). | | | | |
| --- | --- | --- | --- | --- |
|  |  | **β (95%CI)** | **β (95%CI)** | β (95%CI) |
| Poverty | No | Ref | Ref | Ref |
|  | Yes | −0.67^**^ (−0.94 to −0.41) | −0.75^**^ (−1.01 to −0.48) | −0.70^**^ (−0.97 to −0.44) |
| Parental involvement behaviors | Total score (0-14) | 0.26^**^ (0.21 to 0.32) |  |  |
| Parental interaction with child | Total score (0-9) |  | 0.31^**^ (0.25 to 0.37) |  |
| Parental care for child's physical health | Total score (0-5) |  |  | 0.19^**^ (0.07 to 0.31) |
| R^2^ |  | 0.114 | 0.114 | 0.106 |
| All analyses adjusted for child sex, grade, respondent, maternal age, marital status, having old sibling, having young sibling, maternal economic difficulties in childhood, maternal education, all 34 municipalities in Kochi prefecture, caregiver's psychological distress and relationship with neighborhood. ** p<0.01, * p<0.05. | | | | |

| **Table S4.** Association between parental involvement behaviors and young adolescent's self-esteem among adolescents living in poverty (n=3,696). | | | |  |  |
| --- | --- | --- | --- | --- | --- |
|  |  |  |  |  |  |
|  |  | Crude | Model 1^a^ | Model 2^b^ | Model 3^c^ |
| Parental involvement |  | β (95%CI) | β (95%CI) | β (95%CI) | β (95%CI) |
| Parental interaction with child |  |  |  |  |  |
| Helping child study | <1 a week | Ref | Ref | Ref | Ref |
|  | 1+ a week | 1.69^**^ (1.23 to 2.14) | 0.13 (−0.41 to 0.68) | 0.02 (−0.52 to 0.56) | −0.44 (−1.00 to 0.13) |
| Talking about school life | <5 a week | Ref | Ref | Ref | Ref |
|  | Almost everyday | 1.12^**^ (0.70 to 1.55) | 0.88^**^ (0.44 to 1.31) | 0.81^**^ (0.39 to 1.24) | 0.30 (−0.19 to 0.79) |
| Talking about news | <3 a week | Ref | Ref | Ref | Ref |
|  | 3+ a week | 1.13^**^ (0.65 to 1.61) | 1.21^**^ (0.74 to 1.68) | 1.16^**^ (0.70 to 1.63) | 0.80^**^ (0.28 to 1.32) |
| Playing with child (physical activity) | Rarely | Ref | Ref | Ref | Ref |
|  | 1+ a month | 2.21^**^ (1.75 to 2.67) | 1.00^**^ (0.47 to 1.52) | 0.88^**^ (0.36 to 1.41) | 0.82^**^ (0.28 to 1.32) |
| Talking about TV shows with child | <5 a week | Ref | Ref | Ref | Ref |
|  | Almost everyday | 0.51^*^ (0.07 to 0.94) | 0.55^*^ (0.12 to 0.99) | 0.50^*^ (0.07 to 0.93) | −0.12 (−0.61 to 0.37) |
| Cooking with child | <3 a month | Ref | Ref | Ref | Ref |
|  | 1+ a week | 0.39 (−0.11 to 0.88) | 0.34 (−0.15 to 0.84) | 0.28 (−0.21 to 0.77) | −0.16 (−0.68 to 0.35) |
| Going out with child | <3 a month | Ref | Ref | Ref | Ref |
|  | 1+ a week | 1.00^**^ (0.58 to 1.43) | 0.38 (−0.07 to 0.83) | 0.31 (−0.14 to 0.76) | −0.04 (−0.50 to 0.43) |
| Talking about child's future | Sometimes or less | Ref | Ref | Ref | Ref |
|  |  | 1.19^**^ (0.70 to 1.68) | 1.60^**^ (1.12 to 2.08) | 1.50^**^ (1.02 to 1.98) | 1.20^**^ (0.69 to 1.70) |
| Hosting events for child | No | Ref | Ref | Ref | Ref |
|  | Yes | 2.26^**^ (1.07 to 3.44) | 1.80^**^ (0.61 to 3.00) | 1.51^*^ (0.29 to 2.74) | 1.24^*^ (0.03 to 2.45) |
| Parental care for child physical health |  |  |  |  |  |
| Having experience of not visiting the hospital for child | Yes | Ref | Ref | Ref | Ref |
|  | No | 0.42 (−0.15 to 0.99) | 0.56 (−0.03 to 1.11) | 0.42 (−0.14 to 0.98) | 0.48 (−0.08 to 1.04) |
| History of routine vaccination | No/unknown | Ref | Ref | Ref | Ref |
|  | Yes | 0.86^*^ (0.14 to 1.57) | 0.56 (−0.16 to 1.28) | 0.44 (−0.27 to 1.14) | 0.36 (−0.35 to 1.06) |
| Maternal smoking in front of child | Smoking in front of child | Ref | Ref | Ref | Ref |
|  | Never smoking/smoking but not in front of child | 0.38 (−0.09 to 0.86) | −0.03 (−0.51 to 0.45) | −0.05 (−0.52 to 0.42) | −0.12 (−0.62 to 0.37) |
| Paternal smoking in front of child | Smoking in front of child | Ref | Ref | Ref | Ref |
|  | Never smoking/smoking but not in front of child | 0.27 (−0.22 to 0.77) | 0.05 (−0.45 to 0.55) | 0.04 (−0.45 to 0.54) | −0.01 (−0.52 to 0.50) |
| Cooking for child | <6 (days) week | Ref | Ref | Ref | Ref |
|  | Almost everyday | 1.08^**^ (0.55 to 1.61) | 0.72^**^ (0.19 to 1.25) | 0.61^*^ (0.09 to 1.13) | 0.45 (−0.07 to 0.98) |
| ^a^ Adjusted for child sex, grade, respondent, maternal age, marital status, having old sibling, having young sibling, maternal economic difficulties in childhood, maternal education, and all 34 municipalities in Kochi prefecture.  ^b^ Adjusted for caregiver's psychological distress and relationship with neighborhood. ^c^ Included all parental involvements behaviors.  ** p<0.01, * p<0.05. | | | | | |

| **Table S5.** Association between parental involvement behaviors and young adolescent's self-esteem among adolescents living in poverty (n=7,088). | | | |  |  |
| --- | --- | --- | --- | --- | --- |
|  |  | **Crude** | **Model 1^a^** | **Model 2^b^** | **Model 3^c^** |
| **Parental involvement** |  | **β (95%CI)** | **β (95%CI)** | **β (95%CI)** | **β (95%CI)** |
| Parental interaction with child |  |  |  |  |  |
| Helping child study | <1 a week | Ref | Ref | Ref | Ref |
|  | 1+ a week | 2.24^**^ (1.92 to 2.56) | 0.18 (−0.20 to 0.55) | 0.12 (−0.25 to 0.50) | −0.40^*^ (−0.79 to −0.01) |
| Talking about school life | <5 a week | Ref | Ref | Ref | Ref |
|  | Almost everyday | 1.91^**^ (1.62 to 2.20) | 1.43^**^ (1.14 to 1.72) | 1.36^**^ (1.07 to 1.66) | 0.98^**^ (0.64 to 1.32) |
| Talking about news | <3 a week | Ref | Ref | Ref | Ref |
|  | 3+ a week | 1.19^**^ (0.87 to 1.51) | 1.00^**^ (0.69 to 1.31) | 0.94^**^ (0.63 to 1.25) | 0.37^*^ (0.03 to 0.71) |
| Playing with child (physical activity) | Rarely | Ref | Ref | Ref | Ref |
|  | 1+ a month | 2.39^**^ (2.07 to 2.70) | 0.80^**^ (0.45 to 1.14) | 0.70^**^ (0.36 to 1.05) | 0.59^**^ (0.24 to 0.95) |
| Talking about TV shows with child | <5 a week | Ref | Ref | Ref | Ref |
|  | Almost everyday | 0.93^**^ (0.62 to 1.23) | 0.79^**^ (0.50 to 1.09) | 0.74^**^ (0.45 to 1.03) | 0.08 (−0.24 to 0.41) |
| Cooking with child | <3 a month | Ref | Ref | Ref | Ref |
|  | 1+ a week | 0.58 (0.19 to 0.96) | 0.20 (−0.18 to 0.57) | 0.13 (−0.25 to 0.50) | −0.34 (−0.73 to 0.04) |
| Going out with child | <3 a month | Ref | Ref | Ref | Ref |
|  | 1+ a week | 1.37^**^ (1.08 to 1.66) | 0.36^*^ (0.06 to 0.66) | 0.34^*^ (0.04 to 0.64) | 0.02 (−0.29 to 0.33) |
| Talking about child's future | Sometimes or less | Ref | Ref | Ref | Ref |
|  |  | 1.17^**^ (0.84 to 1.50) | 1.40^**^ (1.09 to 1.72) | 1.36^**^ (1.05 to 1.68) | 0.97^**^ (0.64 to 1.30) |
| Hosting events for child | No | Ref | Ref | Ref | Ref |
|  | Yes | 2.78^**^ (0.96 to 4.61) | 1.32 (−0.53 to 3.17) | 1.24 (−0.57 to 3.06) | 1.03 (−0.81 to 2.86) |
| Parental care for child physical health |  |  |  |  |  |
| Having experience of not visiting the hospital for child | Yes | Ref | Ref | Ref | Ref |
|  | No | 0.50^**^ (0.01 to 0.98) | 0.95^**^ (0.49 to 1.41) | 0.84^**^ (0.38 to 1.31) | 0.76^**^ (0.29 to 1.22) |
| History of routine vaccination | No/unknown | Ref | Ref | Ref | Ref |
|  | Yes | 0.78^*^ (0.08 to 1.47) | 0.17 (−0.55 to 0.90) | 0.06 (−0.66 to 0.77) | −0.03 (−0.74 to 0.68) |
| Maternal smoking in front of child | Smoking in front of child | Ref | Ref | Ref | Ref |
|  | Never smoking/smoking but not in front of child | 0.34 (−0.13 to 0.81) | 0.09 (−0.37 to 0.54) | 0.05 (−0.40 to 0.50) | −0.01 (−0.47 to 0.46) |
| Paternal smoking in front of child | Smoking in front of child | Ref | Ref | Ref | Ref |
|  | Never smoking/smoking but not in front of child | 0.49^**^ (0.17 to 0.81) | 0.24 (−0.07 to 0.55) | 0.23 (−0.08 to 0.54) | 0.19 (−0.13 to 0.50) |
| Cooking for child | <6 (days) week | Ref | Ref | Ref | Ref |
|  | Almost everyday | 0.49^*^ (0.07 to 0.92) | 0.11 (−0.30 to 0.52) | 0.04 (−0.37 to0.45) | −0.17 (−0.58 to 0.24) |
| ^a^ Adjusted for child sex, grade, respondent, maternal age, marital status, having old sibling, having young sibling, maternal economic difficulties in childhood, maternal education, and all 34 municipalities in Kochi prefecture.  ^b^ Adjusted for caregiver's psychological distress and relationship with neighborhood. ^c^ Included all parental involvements behaviors.  ** p<0.01, * p<0.05. | | | | | |
